# Quality assessment of economic evaluations of health promotion programs for children and adolescents—a systematic review using the example of physical activity

**DOI:** 10.1186/s13561-015-0071-5

**Published:** 2015-11-24

**Authors:** Katharina Korber

**Affiliations:** 1 Munich School of Management and Munich Center of Health Sciences, Ludwig-Maximilians-University, Ludwigstraße 28/RG, 80539 Munich, Germany; 2 Institute for Health Economics and Health Care Management, Helmholtz Zentrum München (GmbH)—German Research Center for Environmental Health, 85764 Neuherberg, Germany

**Keywords:** Review, Economic evaluation, Physical activity, Children and adolescents, Primary prevention, Health promotion

## Abstract

An increasing number of primary prevention programs aimed at promoting physical exercise in children and adolescents are being piloted. As resources are limited, it is important to ascertain the costs and benefits of such programs. The aim of this systematic review is to evaluate the currently available evidence on the cost-effectiveness of programs encouraging physical activity in children and adolescents and to assess their quality. A systematic review was conducted searching in well established literature databases considering all studies before February 2015. Citation tracking in Google Scholar and a manual search of the reference lists of included studies were used to consolidate this. The fundamental methodological elements of the included economic evaluations were extracted, and the quality of the included studies was evaluated using the Pediatric Quality Appraisal Questionnaire (PQAQ). In total, 14 studies were included. Considering the performance of the economic evaluation, the studies showed wide variation. Most of the studies used a societal perspective for their analyses and discounted costs and effects. The findings ranged from US$11.59 for a person to become more active (cheapest intervention) up to US$669,138 for a disability adjusted life year (DALY) saved (most expensive intervention), with everything in between. Overall, the results of three studies are below a value of US$3061, with one of them even below US$200.00, for the achieved effects. For the other programs, the context-specific assessment of cost-effectiveness is problematic as there are different thresholds for cost-effectiveness in different countries or no clearly defined thresholds at all. There are multiple methodological difficulties involved in evaluating the cost-effectiveness of interventions aimed at increasing physical activity, which results in little consistency between different evaluations. The quality of the evaluations ranged from poor to excellent while a large majority of them was of very good methodological quality. Better comparability could be reached by greater standardization, especially regarding systematic consideration of implementation costs.

## Background

According to the WHO, physical inactivity has been identified as the fourth leading risk factor for global mortality with 6 % of deaths globally [1].

Additionally, in recent years, physical inactivity has been increasing in infancy and adolescence and is expected to continue to increase [2, 3]. Physical inactivity has a significant economic impact on health care systems (see for example [4–6]). Hence, the promotion of healthier patterns of living according to physical activity should have high priority in public health interventions in infancy and adolescence [7].

But, as financial resources are limited, only effective and, ideally, only cost-effective programs should be implemented [8]. Therefore, it is useful to gain an impression of already existing economic evaluations of programs encouraging physical activity in children and adolescents to see what is already done in this field, what the economic results are, and what the quality of these evaluations is. Based on this, the aim of this paper is to give an overview of the economic evaluations of programs encouraging physical activity in children and adolescents and also to assess the quality of these evaluations.

## Review

### Systematic database search

To identify health economic evaluations relevant in answering the review question and gain an overview of already existing national and international results concerning the costs and effects of measures to promote physical activity, a comprehensive systematic review of the literature was conducted. The following databases were used to find relevant literature: PubMed, Web of Science, Centre for Reviews and Dissemination (CRD) Databases (DARE, NHS EED, HTA), EconLit, and Embase.

The search was conducted in February 2015 and contains all results published or listed in the databases up to and including January 2015.

The PICOS scheme [9] was used to concretize the search terms for the review. An overview of the terms is given in Table 1.

**Table 1:** Search terms for database research

	Superior search terms	Inferior search terms
Population	1. Infants and adolescents	All children (0–18 years)
Intervention	2. Physical activity (prevention and/or therapy)	Physical activity, movement, exercise, exercise therapy, motor activity, activity, sport, sports, sedentary behavior
Comparators^a^	–	–
Outcomes	3. Costs	Cost studies, cost study, costs
	4. Effects	Program evaluation, effects, effectiveness
Study design	5. Economic evaluation (combination of 3. and 4., implies the terms that were used for the search in 3. and 4. via “Mesh terms”)	Economic evaluation, economics, cost-effectiveness, evaluation, evaluation studies, cost–benefit analysis

^a^no search was made specifically for comparators as, for encouraging physical activity, as there was no need for the purpose of this systematic review to specify any comparators

The terms were used analogously in all databases, adapted to the given search possibilities of the particular databases. Additionally, Google Scholar was used to retrieve further references as well as a manual search of the reference lists of included studies.

### Inclusion/exclusion criteria

As there are already other reviews that concern themselves with the topic of secondary prevention measures (in which the target group is already overweight or obese) [10], these measures were excluded. However, primary prevention measures for obesity that involve all infants regardless of initial weight were considered as well. Studies in which physical activity was used as a secondary prevention measure for already existing diseases in the target group were also excluded. The target group “infants and adolescents” was an essential criterion of the search. Studies that examine measures for other target groups have therefore not been included in the report. Additionally, only English, German, and French publications were included. Publications on developing countries were omitted. Studies that only looked at effects were also excluded. Also, only original studies were included; reviews and meta-analyses, such as that by Wu et al. [11], were not included in this review.

For this review, all programs that aimed to encourage physical activity or prevent physical inactivity were considered, even if they also focused on other parameters besides physical activity, such as nutrition for example.

### Data extraction

To summarize the fundamental characteristics of the health economic evaluations included in the review, data extraction was performed independently by two reviewers using an adapted version of a data extraction template recommended by the Centre for Reviews and Dissemination [9]. It includes detailed information about major characteristics of the particular studies (author, year, country, study type, study objective, intervention, comparator, study population, setting, perspective, time horizon), the methods used in each study (data sources, data used in economic evaluation, outcomes, costs, discounting, analysis of uncertainty), and the results (outcomes, costs, synthesis of outcomes, and costs). Furthermore, there is a short summary of the authors’ conclusions and information about potential funding.

### Quality assessment of economic evaluations

Several checklists were tested (e.g., Philips et al. [12]) but, for the study question here, the Pediatric Quality Appraisal Questionnaire (PQAQ) was considered to be the most appropriate checklist for quality assessment, as it was developed from the established appraisal checklists and specially constructed for application in assessing the quality of measures for the pediatric population [13].

The PQAQ is a 57-item instrument with a total of 14 domains, of which 13 are scored with values from zero to one (1 = yes, 0.5 = partially, 0 = no/not reported). One domain is for adding additional descriptive information. Each domain corresponds to a key aspect of health economic methodology, such as economic evaluation, comparators, target population, time horizon, perspective, costs and resource use, outcomes, quality of life, analysis, discounting, instrumental analysis, sensitivity analysis, conflict of interests, and conclusions. Within each domain, between two and 10 items are requested. In total, this leads to 10 descriptive and 46 scoreable items and one item for overall quality assessment [13, 14]. The quality assessment was conducted independently by two reviewers. Although it is not the goal of the PQAQ to calculate an overall quality score [13, 14], but just to give a subjective rating of overall quality, an overall score was calculated by the author to legitimate the impression of overall quality. Questions which were not applicable (“N.A.”) were excluded from the overall scoring.

The summary score was calculated using the following formula: [1/(*n – x*)]Σ*i Si* × 100 [15], rounded to the nearest 1 %; *i* = 1, .., *n*, *n* is the number of questions, *x* is the number of questions for which the response was N.A., and *S* is the score for each question. This method has already been used in other approaches adopted by researchers in developing a quality score from checklists (e.g., [16]). For the ranking, the ranges of the achieved scores were chosen by the author (<30 % (worthless), 30 ≤ 45 % (poor), 45 % ≤ 60 % (fair), 60 % ≤ 75 % (good), 75 %–90 % (very good), >90 % (excellent)). For a detailed calculation of the scores see Additional files Table S4.

For a global impression of the quality of the article, not only the scores were taken into account, but also the information that was given by the qualitative items in the PQAQ.

### Data synthesis

The results of the economic evaluations were adjusted for country-specific inflation. The Consumer Price Indices (CPI) as part of the Main Economic Indicators (MEI) of the OECD [17, 18] and the purchasing power parity (PPP) conversion rate of 2011 were used to convert monetary results to U.S. dollars [19], as this was the price year of the most recent intervention.

Detailed information on the results of the quality assessment are given in Table 3.

## Results

### Results of the research

**Fig. 1:**
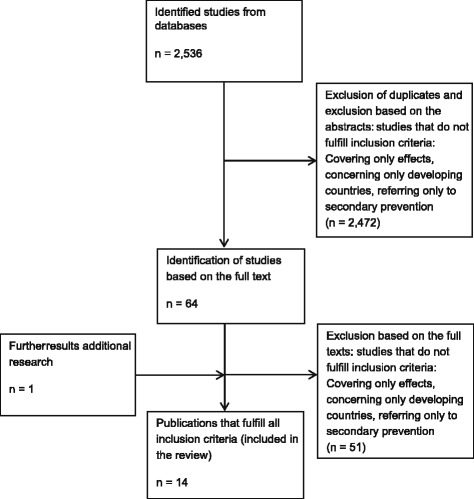
Flow chart for selection of economic evaluations

Figure 1 shows the number of hits and the selection process for the relevant studies.

In total, 14 studies were found that fulfilled the inclusion criteria. Thirteen different interventions to increase physical activity in children and adolescents (and to some extent better nutrition and a healthier lifestyle in general) were examined with regard to the prevention of overweight and obesity. One of the interventions was also examined with regard to the prevention of Disordered Weight Control Behavior (DWCB).

### Characteristics of the economic evaluations

Table 2 gives an overview of the characteristics of the 14 relevant economic evaluations and concludes the key economic findings (for the base case scenarios). Details of the economic findings and the study characteristics can also be found in Korber [20], a review focusing on the transferability of economic evaluations which was updated here for lying the focus on the quality of the evaluations.

**Table 2:** Study description and key economc findings (alphabetically sorted)

Author/Year/(Country)	Intervention components	Target/Age group	Setting	Study design	Perspective, time horizon, discounting	Measure of effects	Price Year/Currency unit, considered cost categories	Result [in 2011 US$]*
Brown et al. 2007 (USA) [23]	Physical activity, nutrition	Children, grades three, four and five, 8–11 years	School	CUA, using a model approach (calculating additional benefit)	Society, modeling over a 25-year period, costs and benefits at 3 %	Cases of adult overweight prevented (40–64 years), QALYs saved	2004, US$, intervention costs, avoided treatment costs, avoided productivity loss costs	Cost per QALY saved = US$ 900 [US$ 1072.52], Net benefit = US$ 68,125 [US$ 81,183.60]
Kesztyüs et al. 2011 (GER) [26]	Health education, physical activity breaks, and parent involvement	Children, primary school, second grade, 7–8 years	School	CEA, using intervention results	Society, 1 year, not stated	Differences in waist-to-height ratio, waist circumference, and BMI,	2008, EUR, total intervention costs, intervention costs per child	ICER (WC) = EUR 11.11 [US$ 14.67] per cm prevented
								ICER (WHtR) = EUR 18.55 [US$ 24.50] per unit prevented
Krauth et al. 2013 (GER) [28]	3 additional PE lessons per week	Children, primary school	School	CEA, using intervention results	Society, not stated, not stated	Reduction in BMI, increase in physical activity	No price year, EUR, intervention costs, intervention costs per child per school year	EUR 619/child/year for significant results [US$ 789.54]
McAuley et al. 2010 (NZ) [29]	Nutrition and physical activity	Children, 5–12 years	School/Community	CEA, using intervention results	Society, 4 years, costs at 5 %	Weight gain avoided, QALY	2006, NZ$, no development costs, total costs	NZ$ 664–1708 [US$ 515.53–1326.1] per kg avoided weight gain (depending on age), no QALY gain reported
Moodie et al. 2009 (AUS) [22]	“Walking School Bus” encouraging physical activity	Children, 5–7 years	School/Community	CUA, using a model approach	Society, lifetime, costs and benefits both at 3 %	Reduction in BMI, increase in physical activity, energy expenditure	2001, AU$, total costs	Lifetime DALYs, Cost per: DALY saved: AU$ 760,000 [US$ 669,138.39] (net, gross: AU$ 770,000 [US$ 677,942.84])
								- BMI unit saved: AU$ 87,000 [US$ 76,598.74]
Moodie et al. 2010 (AUS) [34]	After-school care for children from 3 to 5 pm including a physical activity program	Children, primary school, 5–11 years	School	CUA, using a model approach	Society, lifetime, costs and benefits both at 3 %	Reduction in BMI, increase in physical activity, energy expenditure	2001, AU$, total cost	Lifetime DALYs, Gross cost per:
								- DALY saved: AU$ 82,000 [US$ 72,196.51] (net, gross: AU$ 90,000 [US$ 79,240.07]
								- BMI unit saved: AU$ 8200 [US$ 7219.65]
Moodie et al. 2011 (AUS) [35]	Lessons, information evenings, promotion of the program	Children, 5th and 6th school years, 10–11 years	School/Community	CUA, using a model approach	Society, lifetime, costs and benefits both at 3 %	Reduction in BMI, increase in physical activity, energy expenditure, DALY	2001, AU$, total costs	Lifetime DALYs, Cost per:
								- DALY saved: AU$ 117,000 [US$ 103,012.09] (net, gross: AU$ 125,000 [US$ 110,055.66])
								- BMI unit saved: AU$ 13,000 [US$ 11,445.79]
Moodie et al. 2013 (AUS) [30]	Interdisciplinary approach, including nutrition and physical activity and reducing screen time	Children, 4–12 years	School/Community	CUA, using a model approach	Society, lifetime, costs and benefits both at 3 %	Reduction in BMI, DALY	2006, AU$, total costs	Lifetime DALYs, Cost per:
								- DALY saved: AU$ 20,227 [US$ 15,478.09] (net, gross: AU$ 22,978 [US$ 17,583.21])
								- BMI unit saved: AU$ 399 [US$ 305.32]
Peterson et al. 2008 (USA) [21]	Media campaign	Teenagers, 12–17 years	Society	CEA, using intervention results	Only program costs, not reported, not stated	Questionnaire, extrapolated to population: “contemplated doing more exercise”, “has done more exercise”	No price year, US$, development costs of the program and costs for “product placement”	Cost per person who did more exercise: between US$ 5.11 [US$ 6.68] and US$ 153.19 [US$ 200.12] for the individual sections of the campaign, US$ 8.87 [US$ 11.59] for the whole campaign
Pringle et al. 2010 (UK) [24]	Activity classes, free swimming activities	Population (children 10–17 years)	Community	CUA, using a model approach	Key implementation and running costs, not stated, not stated	Change in MPA, QALY	2003, £, costs/completer improving MPA	Cost per QALY gained
								- Activity: £ 94 [US$ 166.07]
								- Swimming: £ 103 [US$ 181.97]
								NHS savings per completer
								- Activity: £ 769 [US$ 1358.59]
								- Swimming: £ 2111 [US$ 3729.49]
Rush et al. 2014 (NZ) [37]	Multicomponent through-school physical activity and nutrition program	Primary school children, 6–8 and 9–11 years	School	CUA, using a model approach	Funder’s perspective, lifetime, costs and outcomes both at 3.5 %	QALY, increased life expectancy.	2011, NZ$, lifetime costs, incremental costs	ICER/QALY (older children): NZ$ 24,690 [US$ 16,570.47], ICER/QALY (younger children): NZ$ 30,438 [US$ 20,428.19]
						Existing model used to extrapolate the effects and costs		
Wang et al. 2003 (USA) [36]	Interdisciplinary approach, lessons, sport materials, wellness, teacher training	Children 6th–8th school year, 11–13 years	School	CUA, using a model approach (calculating additional benefit)	Society, modeling over a 25-year period, costs and benefits both at 3 %	Cases of adult overweight prevented (5.805), QALYs (4.13)	1996, US$, intervention costs, avoided treatment costs, avoided productivity loss costs	Cost per QALY saved: US$ 4305 [US$ 6179.08], Net benefit: US$ 7313 [US$ 10,496.55]
Wang et al. 2008 (USA) [27]	After school program: physical activity, healthy snacks, support with homework, and “academic enrichment”	Children, Elementary school, 6–10 years	School	CEA, using intervention results	Society, 1 year, not stated	% Reduction in body fat	2003, US$, intervention costs, after-school care costs without intervention	US$ 417 [US$ 509.89] per % point body fat reduction
Wang et al. 2011 (USA) [25]	Interdisciplinary approach, lessons, sport materials, wellness, teacher training	Children (6th–8th school year), 11–13 years	School	CUA, using a model approach (calculating additional benefit)	Society, 10 years, costs and benefits both at 3 %	DWCB avoided, QALYs	2010, US$, total costs	Cost per QALY saved (DWCB and obesity combined) = US$ 2966 [US$ 3060.91], net benefit (DWCB + obesity) = US$ 14,238 [US$ 14,693.62]

*Results were adjusted to the year 2011 (year of the study with the newest data) using consumer price index (CPI) as part of the Main Economic Indicators (MEI) of the OECD and purchasing power parity (PPP) conversion rate of the year of the latest intervention to convert numerical results to U.S. dollars

*AU$* Australian dollar, *AUS* Australia, *CEA* cost-effectiveness analysis, *CUA* cost–utility analysis, *DALY* disability adjusted life year, *DWCB* disordered weight control behaviors, *EUR* Euro, *£* Great British pound, *MPA* moderate physical activity, *NHS* national health service, *NZ* New Zealand, *NZ$* New Zealand dollar, *QALY* quality adjusted life year, *USA* United States of America, *US$* U.S. dollar, *WC* waist circumference, *WHtR* waist-to-height ratio

As can be seen in Table 2, the results of the economic evaluations showed a wide range of values with different outcomes which are not directly comparable (e.g., “becoming more active”, QALY, DALY). Taking the value in US$ for the price year 2011, which was calculated based on the original publications, the lowest value was US$8.78 in the year 2000 (US$11.59 in 2011) [21] for a person to become more active because of a media campaign. The highest value was up to AU$760,000 in the year 2001 (US$669,138 in 2011) for a DALY saved by a “Walking School Bus” program [22]. The other programs ranged between these values.

The economic results of three of the studies presented in this review are below a value of US$2966/QALY saved (US$3061 for the year 2011) [23–25]. One of these studies even shows a value below £94/QALY saved or £103/QALY saved in 2003 (US$200 in 2011) [24], which can be assumed to be a very low value even for prevention/health promotion measures.

### Quality of the economic evaluations

Table 3 gives a detailed overview of the quality assessment for the 14 relevant studies, sorted in alphabetical order according to the PQAQ. As can be seen, there is a wide range in the overall quality of the studies ranging from “poor” to “excellent” with a majority of studies showing very good quality. As the results for the quality assessment are presented in detail in Table 3 and in the additional documents only some notable results are described in the following.

**Table 3:** Quality assessment of economic evaluations—overview

		Brown et al. (2007) [23]	Kesztyüs et al. (2011) [26]	Krauth et al. (2013) [28]	McAuley et al. (2010) [29]	Moodie et al. (2009) [22]	Moodie et al. (2010) [34]	Moodie et al. (2011) [35]	Moodie et al. (2013) [30]	Peterson et al. (2008) [21]	Pringle et al. (2010) [24]	Rush et al. (2014) [37]	Wang et al. (2003) [36]	Wang et al. (2008) [27]	Wang et al. (2011) [25]
Economic evaluation	Is the research question posed in terms of costs and consequences?	1	1	1	1	1	1	1	1	1	1	1	1	1	1
	Is a specific type of economic analysis technique performed?	1	1	0.5	1	1	1	1	1	1	1	1	1	1	1
	Domain Score	2	2	1.5	2	2	2	2	2	2	2	2	2	2	2
Comparators	Is there a rationale for choosing the intervention(s) being investigated?	1	1	1	1	0.5	0.5	1	1	1	1	1	1	1	1
	Is there a rationale for choosing the alternative program(s) or intervention(s) used for comparison?	0.5	0.5	0.5	0.5	0.5	0.5	0.5	0.5	0	0.5	0.5	0.5	0.5	0.5
	Does the report describe the alternatives in adequate detail?	0.5	1	0.5	1	0.5	0.5	0.5	0.5	0	0	0.5	0.5	0.5	0.5
	Is a description of the event pathway provided?	1	N.A.	N.A.	N.A.	0.5	0	0.5	0.5	N.A.	0	0.5	1	N.A.	0.5
	Is a formal decision analysis performed?	0.5	N.A.	N.A.	N.A.	0	0	0	0.5	N.A.	0	0	0.5	N.A.	0
	Domain Score	3.5	2.5	2	2.5	2	1.5	2.5	3	1	1.5	2.5	3.5	2	2.5
Target population	Is the target population for the intervention identified?	1	1	1	1	1	1	1	1	0.5	0.5	1	1	1	1
	Are the subjects representative of the population to which the intervention is targeted?	1	1	1	1	1	1	1	1	0.5	0.5	1	1	1	1
	Domain Score	2	2	2	2	2	2	2	2	1	1	2	2	2	2
Time horizon	Is there a time horizon for both costs and outcomes?	1	1	1	1	1	1	1	1	1	0.5	1	1	1	1
	Do the authors justify the time horizon selected?	0.5	0.5	0.5	0.5	0.5	0.5	0.5	1	0.5	0.5	0.5	0.5	0.5	0.5
	Domain Score	1.5	1.5	1.5	1.5	1.5	1.5	1.5	2	1.5	1	1.5	1.5	1.5	1.5
Perspective	Is a perspective for the analysis given?	1	1	1	1	1	1	1	1	0	0	1	1	1	1
	Is a societal perspective taken, either alone or in addition to other perspectives?	1	1	1	1	1	1	1	1	0	0	0	1	1	1
	When there is more than one perspective, are the results of each perspective presented separately?	N.A.	N.A.	N.A.	N.A.	N.A.	N.A.	N.A.	N.A.	N.A.	N.A.	N.A.	N.A.	N.A.	N.A.
	Domain Score	2	2	2	2	2	2	2	2	0	0	1	2	2	2
Costs and resource use	Are all relevant costs for each alternative included?	1	1	1	1	1	1	1	1	1	1	1	1	1	1
	Are opportunity costs of lost time (productivity costs) for parents and informal caregivers measured when required?	N.A.	N.A.	1	N.A.	N.A.	N.A.	N.A.	1	N.A.	N.A.	N.A.	N.A.	N.A.	N.A.
	Do cost item identification and valuation extend beyond the health-care system to include school and community resources when necessary?	1	1	1	1	1	1	1	1	0.5	1	0.5	1	1	1
	Are future salary and productivity changes of the child taken into consideration when appropriate?	1	N.A.	N.A.	N.A.	0	0	0	0	N.A.	0	N.A.	1	N.A.	0
	Are all of the sources for estimating the volume of resource use described?	1	1	0.5	0.5	1	1	1	1	0	0	0.5	1	1	0.5
	Are all the sources for estimating all of the unit costs described?	1	1	0.5	0.5	1	1	1	1	0	0.5	0.5	0.5	1	0.5
	Domain Score	5	4	4	3	4	4	4	5	1.5	2.5	2.5	4.5	4	3
Outcomes	Is a primary health outcome given?	0.5	1	1	1	0.5	0.5	0.5	1	0	0	1	0.5	1	0.5
	Do the authors justify the health outcome(s) selected?	1	1	0.5	1	1	1	1	1	1	1	0.5	1	1	1
	Is effectiveness, rather than efficacy, assessed?	1	1	1	1	1	1	1	1	1	1	1	1	1	1
	Are the details of the design of the effectiveness/efficacy study(s) provided?	1	1	1	1	1	1	1	1	0.5	0.5	1	1	0.5	0.5
	Are the results of the efficacy/effectiveness of alternatives reported?	1	1	1	1	0.5	0.5	1	1	1	0.5	0.5	1	1	0.5
	Are school/day-care absences taken into consideration?	N.A.	N.A.	N.A.	N.A.	N.A.	N.A.	N.A.	N.A.	N.A.	N.A.	N.A.	N.A.	N.A.	N.A.
	If intermediate outcome variables are used, are they linked by evidence or reference to the end benefit?	1	1	N.A.	N.A.	1	1	0.5	0.5	N.A.	0.5	0.5	0.5	1	0.5
	Domain Score	5.5	6	4.5	5	5	5	5	5.5	3.5	3.5	4.5	5	5.5	4
Analysis	Are costs AND outcomes measured in units appropriate for the indicated analytic technique?	1	1	0.5	1	1	1	1	1	0.5	1	1	1	1	1
	Are costs valued appropriately?	1	1	1	1	1	1	1	1	1	1	1	1	1	1
	Is the valuation of outcomes appropriate for the type of analysis?	1	1	0.5	1	1	1	1	1	0.5	0.5	1	1	1	1
	Are quantities of resources used reported separately from their unit costs?	1	1	0.5	1	1	0.5	0.5	0.5	0	0.5	0	1	1	1
	Are the costs aggregated correctly?	1	1	0.5	1	0.5	0.5	0.5	0.5	0	0.5	0.5	0.5	1	1
	Are details of statistical tests and confidence intervals given for stochastic data?	1	1	0	1	0.5	1	1	1	0	0	1	1	0.5	1
	Domain Score	6	6	3	6	5	5	5	5	2	3.5	4.5	5.5	5.5	6
Discounting	When required, are costs and consequences that occur over more than 1 year discounted to their present values?	1	N.A.	N.A.	0.5	1	1	1	1	N.A.	0	1	1	N.A.	1
	If costs or benefits are not discounted when the time horizon exceeds 1 year, is an explanation provided?	N.A.	N.A.	N.A.	0	N.A.	N.A.	N.A.	N.A.	N.A.	0	N.A.	0	N.A.	N.A.
	Domain Score	1	N.A.	N.A.	0.5	1	1	1	1	N.A.	0	1	1	N.A.	1
Incremental analysis	Are incremental estimates of costs and outcomes presented?	1	1	0.5	0.5	1	1	1	1	0.5	0.5	1	1	1	1
	Are the incremental estimates summarized as incremental ratios?	1	1	N.A.	0	1	1	1	1	0	0	1	1	0.5	1
	Are confidence intervals/limits calculated for incremental ratios or incremental estimates of costs and outcomes?	0	1	0	1	1	1	1	1	0	0	1	0.5	0	1
	Domain Score	2	3	0.5	1.5	3	3	3	3	0.5	0.5	3	2.5	1.5	3
Sensitivity analysis	Are all important assumptions given?	1	0.5	1	0.5	1	1	1	1	0	0	0.5	1	1	1
	Is a sensitivity analysis performed?	1	1	0.5	1	1	1	1	1	0	1	1	1	1	1
	Do the authors justify the alternative values or ranges for sensitivity analysis?	1	0	0.5	0.5	1	1	1	0.5	N.A.	0	0.5	1	1	0.5
	Domain Score	3	1.5	2	2	3	3	3	2.5	0	1	2	3	3	2.5
Conflict of interest	Does the article present the relationship with the sponsor of the study?	1	1	1	1	1	1	1	1	0	0.5	1	1	0	1
	Does the article indicate that the authors had independent control over the methods and right to publish?	0.5	0.5	1	0.5	1	1	1	1	0.5	1	0	0.5	0.5	1
	Domain Score	1.5	1.5	2	1.5	2	2	2	2	0.5	1.5	1	1.5	0.5	2
Conclusions	Is the answer to the study question provided?	1	1	1	0.5	1	1	1	1	1	1	1	1	1	1
	Are the most important limitations of the study discussed?	1	1	1	1	1	1	1	1	1	1	1	1	1	1
	Do the authors generalize the conclusions to other settings or patient/client groups?	0.5	1	0.5	0.5	0.5	0.5	0.5	0.5	0.5	0.5	0.5	1	0.5	0.5
	Domain Score	2.5	3	2.5	2	2.5	2.5	2.5	2.5	2.5	2.5	2.5	3	2.5	2.5
Overall score for quality assessment in percent		89	92	74	81	83	82	85	87	43	48	73	86	84	81
Global impression of the quality of the article		Very good	Excellent	Good	Very good	Very good	Very good	Very good	Very good	Poor	Fair	Good	Very good	Very good	Very good

As there are five studies [21, 26–29] using intervention results, all the questions dealing with the quality of models had to be answered with “N.A.” Furthermore, none of the studies took more than one perspective and in the section “Outcomes” the question of considering school/day care absence was also not applicable for the studies. Opportunity costs for parents and informal caregivers were only measured in two studies [28, 30] while these costs were not applicable in all the other studies (e.g., encouragement of physical activity during school time or regular after school care already included in salaries).

As can be seen many possible scoring points were lost in the questions concerning comparators. The rationale for choosing alternatives used for comparison was made only implicitly by all studies except one [21]. Also for the question of the description of the alternatives in detail only two studies fully scored [26, 29], two did not score at all [21, 24] and the others scored 0.5. Additionally most of the studies for which it was applicable lost points by not adequately describing the pathway provided and even more points were lost by not (adequately) performing formal decision analysis.

## Discussion

### Major findings

Concerning the “effectiveness of projects for physical activity in infants and adolescents”, a large number of studies were found, and there are also detailed literature reviews on these (see for example [31–33]). Looking at cost-effectiveness, in contrast, the situation is completely different.

#### Economic evaluation

Some programs only considered the health effects of the programs in their calculations, other possible positive side-effects that might occur, such as the encouragement of social cohesion (see, for example, [34]) or fewer accidents because of synergy effects with road safety education (which could result from the “Walking Bus” concept from [22], for example), were discussed but not taken into account [22, 34]. Considering these positive side-effects as well could potentially change the cost-effectiveness of these interventions. Furthermore, the economic evaluation of some of the interventions resulted from one project (ACE-Obesity project), which means that they are all based on the same assumptions and modeling techniques. Changes to those rather conservative assumptions [22, 34, 35] could also give a different view of the cost-effectiveness of the programs.

Four studies only used intervention results and a relatively short time horizon [21, 26, 27, 29]. They do not report a gain in QALY or DALY, but they report relatively low costs for achieving health-relevant effects such as reduction in body fat or waist circumference.

Looking at the methodology of the studies the comparability is limited as the studies use different forms of economic evaluations (CUA [22–25, 30, 34–37], CEA [21, 26–29]) with (the resulting) different outcomes (QALY, DALY, BMI reduction, reduction in waist circumference,…). Regarding the different outcomes one major point influencing the implementation of a program is, what e.g., decision makers or funders of programs are willing to pay for the outcome reached by a measure as reaching a QALY is quite different from “becoming more active”.

Additionally different methods of evaluation were used. Some studies used modeling based on different data sources [22–25, 30, 34–37], while others used the intervention data for generating their results [21, 26–29].

Regarding the cost-effectiveness of the programs, it also has to be taken into account, that the context-specific assessment of cost-effectiveness is problematic as there are different thresholds for cost-effectiveness in different countries (e.g., AU$50,000 in Australia; see, for example, [22]) or no clearly defined thresholds at all.

Yet another difficulty results from the fact, that some of the programs considered in this review are complex interventions and not only encouraging physical activity (e.g., combined with the encouragement of better nutrition (see for example [23, 27, 29, 36]). Therefore it is not always clear which effects explicitly result from the encouragement of physical activity.

#### Quality assessment

The most positive aspect of the quality assessment is that none of the studies was ranked as “worthless”. Only one study ranked “poor”, one “fair”, two “good”, nine studies “very good”, and one “excellent”. This shows that there is an awareness of the necessity for high-quality economic evaluations. But it also shows that there is still room for improvement. One problem regarding the methodological shortcomings that led to a loss of points in the quality assessment could be the fact that the quality had to be assessed based on the published data for the studies. This means there could be a deviation between what has been reported about the economic evaluation and what has actually been done, but is not reported. This would not be a lack of quality but rather a lack of transparency.

### Comparison with other reviews

Reviews focusing on the effectiveness of physical activity programs [31–33] show diverse results for the effectiveness of different kinds of program (see also [31–33]). Van Sluijs et al. found strong evidence that school-based interventions can increase physical activity in adolescents and Camacho-Minano et al. found multicomponent school-based interventions that also offer physical education that address the needs of girls as effective [33]. Kahn et al. Also found some school-based programs tob e effective while for others no conclusion about the effectiveness can be made, because of lacking information [32]. The studies also criticize the still missing quality in the evaluations [31, 33].

There is also a review by Wu et al. that converts the effects of physical activity interventions measured in metabolic equivalents (METs) to make them comparable and calculates the cost-effectiveness of these interventions [11]; however, it does not explicitly focus on interventions for children and adolescents. One current review looked at the potential monetary savings of different school-based programs for increasing physical activity, but not at the costs necessary to reach these effects [38]. There are also reviews on economic evaluations of physical activity programs as primary prevention methods in adults [8], and there are several reviews of economic evaluations focusing on physical activity as a (disease-specific) secondary prevention method for both children/adolescents (for example, for obesity: [39]) and adults (for example, reduction of risk factors for metabolic syndrome: [40]). There is also one review focusing on the transferability of economic evaluations of physical activity programs for children and adolescents [20], which overlaps in part with the review conducted here. A reason for that might be that high transparency in presenting the methods of a study leads to high scoring in quality assessment as well as in transferability assessment. But the main difference in the two reviews is that the other one focuses on transferability assessment, whereas this one fills the gap of missing overviews concerning the quality assessment of economic evaluations in this research field.

### Limitations of this review

One limitation of this review is that the collection of publications was limited to those referenced in the databases PubMed, Web of Science, CRD Databases (DARE, NHS EED, HTA), EconLit, and Embase. Citation tracking in Google Scholar and an additional manual search were used to broaden the search. A further restriction resulted from including only publications in English, German, and French, excluding publications in other languages. Only publications up to February 2015 are captured in this review. There always is the problem, that the selection and assessment of relevant studies is not completely objective. Therefore in this review, the screening for relevant publications was made in two steps (first step: only abstracts, second step: full texts, see Fig. 1) by two independent reviewers to achieve higher objectivity.

The PQAQ was used for the quality appraisal of the economic evaluations. It is a validated tool for the assessment of economic evaluations focusing on children [14]. Still, it was sometimes difficult to assess study quality. There is a highly contentious debate in the literature about using quality scoring systems [9]. Nevertheless, overall scoring was made to support the estimation of overall quality. Additionally, the qualitative appraisal items in the PQAQ were taken into account in the overall quality decision. Other checklists were tested (e.g., Philips et al. [12]) but, for the study question here, the PQAQ was considered as the most appropriate checklist for quality assessment. The quality assessment and the underlying data extraction has been conducted independently by two reviewers. Conflicting results in the assessments were discussed and resolved by consensus. The costs of the studies were adjusted to U.S. dollars for the year 2011 by using the CPI [17] and the purchasing power parity (PPP) conversion rate of 2011 [19], to achieve more comparability between studies. However, the explanatory power of those values is limited, e.g., because country-specific health care systems with different pricing schemes were not taken into account in the calculation. Furthermore, health care costs do not necessarily increase in the same way as CPI. There is evidence that health care costs increase even more than CPI. Therefore, the calculation made here tends to be a conservative calculation of the costs [41]. One further general limitation of this review can be seen in the problem of transferability of the results. This arises from the difficulty in assessing behavior-oriented prevention in different settings.

## Conclusions

An essential result of this research is the fact that this is the first review to look at the quality of economic evaluations of programs encouraging physical activity in children and adolescents.

Despite an intensive review of the literature, only a few studies on the economics of programs encouraging physical activity in children and adolescents were found. Looking at the publication years, it can be seen that the majority of the studies found in this research derive from the year 2007 onwards (only one study had already been published in 2003 [36]), giving the impression that this is an ongoing topic in public health research. It becomes apparent that demand for economic evaluations of primary prevention interventions [42] is still not so common in current practice. But looking at the list of excluded studies (see Additional files Table S5), it can be seen that there are several study protocols planning economic evaluations of programs encouraging physical activity in children and adolescents, which gives an interesting perspective for further review updates.

Consideration of the available publications reveals that very different programs have been evaluated and that the results concerning cost-effectiveness are also quite different.

Moreover, attention should be paid to the fact that the individual programs and the results of the economic evaluations are difficult to compare. As can also be seen in the results of this review, the studies differ with regard to their endpoints (e.g., QALYs vs. DALYs), their settings (e.g., school, community, or a combination of both), the date, the intervention components (physical activity only, physical activity and nutrition, physical activity, nutrition, and knowledge transfer), and the thematic organization of these components.

It is also important to point out that at least one of the interventions that was classified as not cost-effective by the authors in the economic evaluation is still being continued and is obviously seen as being successful: the “Australian After-School Communities Program” has far exceeded the assumptions used in the modeling [34]. This example shows that an economic analysis can be a starting point for decision support (and also should be), but that it cannot be the sole basis for a decision because of the above-mentioned complex methodological problems, especially in the area of encouragement of physical activity. During the research, it was also found that an important step toward more (economic) transparency would be documentation of the costs of development, implementation, and continuation of an intervention. Only then can decision makers make at least a rough estimate of what costs would be incurred in a roll-out of the intervention.

Finally, one of the main conclusions that can be drawn from this review is, that even though there are still not many studies in this field (more are planned), but the majority of them shows high or very high quality. The second important conclusion is, that often only low monetary effort is needed to achieve a significant change in physical activity in early age.
